# Rabies vaccine and immunoglobulin supply and logistics: Challenges and opportunities for rabies elimination in Kenya

**DOI:** 10.1016/j.vaccine.2019.05.035

**Published:** 2019-07-17

**Authors:** Gati Wambura, Athman Mwatondo, Mathew Muturi, Carolyne Nasimiyu, Diorbhail Wentworth, Katie Hampson, Philet Bichanga, Collins Tabu, Samuel Juma, Isaac Ngere, SM Thumbi

**Affiliations:** a Center for Global Health Research, Kenya Medical Institute of Research, Kisumu, Kenya; b Zoonotic Disease Unit, Ministry of Health and Ministry of Agriculture, Livestock and Fisheries, Nairobi, Kenya; c The Institute of Biodiversity, Animal Health and Comparative Medicine, University of Glasgow, Glasgow, UK; d Ministry of Health, County Government of Makueni, Kenya; e National Vaccines and Immunization Program, Ministry of Health, Nairobi, Kenya; f Washington State University – Global Health Program, Nairobi, Kenya; g Rabies Free Africa, Washington State University, Pullman, WA, USA; h Paul G. Allen School for Global Animal Health, Washington State University, Pullman, USA

**Keywords:** Rabies, Vaccines, PEP, Immunoglobulins, Kenya

## Abstract

Prompt provision of post-exposure-prophylaxis (PEP) including vaccines and rabies immunoglobulin (RIG) to persons bitten by suspect rabid dogs is a key strategy to eliminating human deaths from dog-mediated rabies in Kenya by 2030. We assessed the availability, forecasting and supply chain logistics for rabies PEP in Kenya, compared with the system used for vaccines in the expanded program of immunization (routine vaccines). Semi-structured questionnaires capturing data on forecasting, procurement, distribution, cold chain and storage, monitoring and reporting for routine vaccines and rabies vaccines and RIG were administered to 35 key personnel at the national, county, sub-county and health facility levels in five counties. Results showed large variability in PEP availability (stockouts ranged from 3 to 36 weeks per year) with counties implementing rabies elimination activities having shorter stockouts. PEP is administered intramuscularly using the 5-dose Essen regimen (day 0, 3, 7, 14 and 28). PEP costs to bite patients were reported to range from 10 to 15 US dollars per dose; RIG was seldom available. A less robust supply and logistics infrastructure is used for rabies PEP compared to routine vaccines. Forecasting and monitoring mechanisms for rabies PEP was poor in the study counties. The supply of vaccines from the national to the sub-national level is mainly through two government agencies and a private agency. Since government decentralization, the National Vaccine and Immunization Program has remained as the main supplier of the routine vaccines, playing a lesser role in the supply of rabies bio-logicals. Adoption of the dose-saving intradermal route for PEP administration, reduction of PEP costs to patients, and placing rabies vaccines within the routine vaccines supply and logistics system would significantly improve PEP availability and accessibility to persons at risk of rabies; a critical step to achieving elimination of human deaths from rabies.

## 1 Introduction

The global target for the elimination of dog-mediated human rabies supported by the World Health Organization and partners has been set for 2030 [1]. The feasibility of this goal, including in Africa and Asia where most of the estimated 59,000 annual human rabies deaths occur, is supported by the existence of potent biologicals for humans and animals, and relatively well understood epidemiology of the disease with domestic dogs the primary reservoir of the virus and source of human infections [2–6].

The strategies for achieving zero human deaths from dog rabies are hinged on two complementary interventions. The first is interrupting transmission between domestic dogs thereby reducing dog-to-human transmission. Control and elimination of dog rabies through mass dog vaccinations has been successful in different settings previously endemic for rabies [7–9]. The second intervention is directly reducing the risk for human rabies, through pre-exposure vaccination for high-risk groups and prompt post-exposure prophylaxis (PEP) that includes wound washing, administration of rabies vaccines, and where indicated, infiltration of rabies immunoglobulin (RIG) into and around the wound(s) [10,11].

Rabies vaccines have been improved over the last few decades, from nerve tissue vaccines that could induce severe adverse reactions to the safer and highly immunogenic cell culture vaccines mostly in use today [11,12]. However, prompt provision of PEP remains a challenge in settings with high incidence of dog bites and dog rabies, leading to unnecessary and preventable human deaths. This has been attributed to the failure of health care systems in rabies endemic regions to ensure steady availability of PEP for bite patients that seek care; socio-economic challenges where bite patients cannot afford the cost of PEP or suffer delays in receiving PEP owing to long distances they have to cover to access health care, or poor health care seeking by bite patients due to a lack of knowledge about the risk of rabies and its prevention [13–15].

In Kenya, rabies is endemic and has been reported in the country for more than a hundred years [16]. In 2014, Kenya launched a 15-year strategy to end human deaths from dog rabies by 2030. The strategy uses a progressive reduction in rabies risk, starting elimination programs in five pilot counties (Siaya, Kisumu, Makueni, Kitui, Machakos) before extending to neighbouring counties until the country is free of rabies [16,17]. The elimination program is focused on mass dog vaccinations, prompt provision of PEP, public health education and awareness on rabies, strengthening rabies surveillance, and operational research to aid in optimal delivery of these interventions. In 2010, Kenya adopted a new constitution that changed governance from a central government system to a devolved system consisting of two levels of government: national government and 47 county governments. As a result, in 2012 health services were devolved to be financed and run by the county governments. Although rabies elimination activities have commenced in parts of the country, there is little data on PEP and RIG availability and accessibility in counties and nationally.

This study addresses the strategy of prompt provision of PEP for patients bitten by suspect rabid dogs. We assessed the rabies vaccine infrastructure including the logistics flow, demand, and supply and forecasting for PEP and RIG at the national, county and sub-county levels. We compared it with the system used for routine vaccines to identify the challenges and opportunities for improving the availability, accessibility, and affordability of rabies PEP to achieve elimination of human rabies in the country.

## 2 Methods

**Fig. 1:**
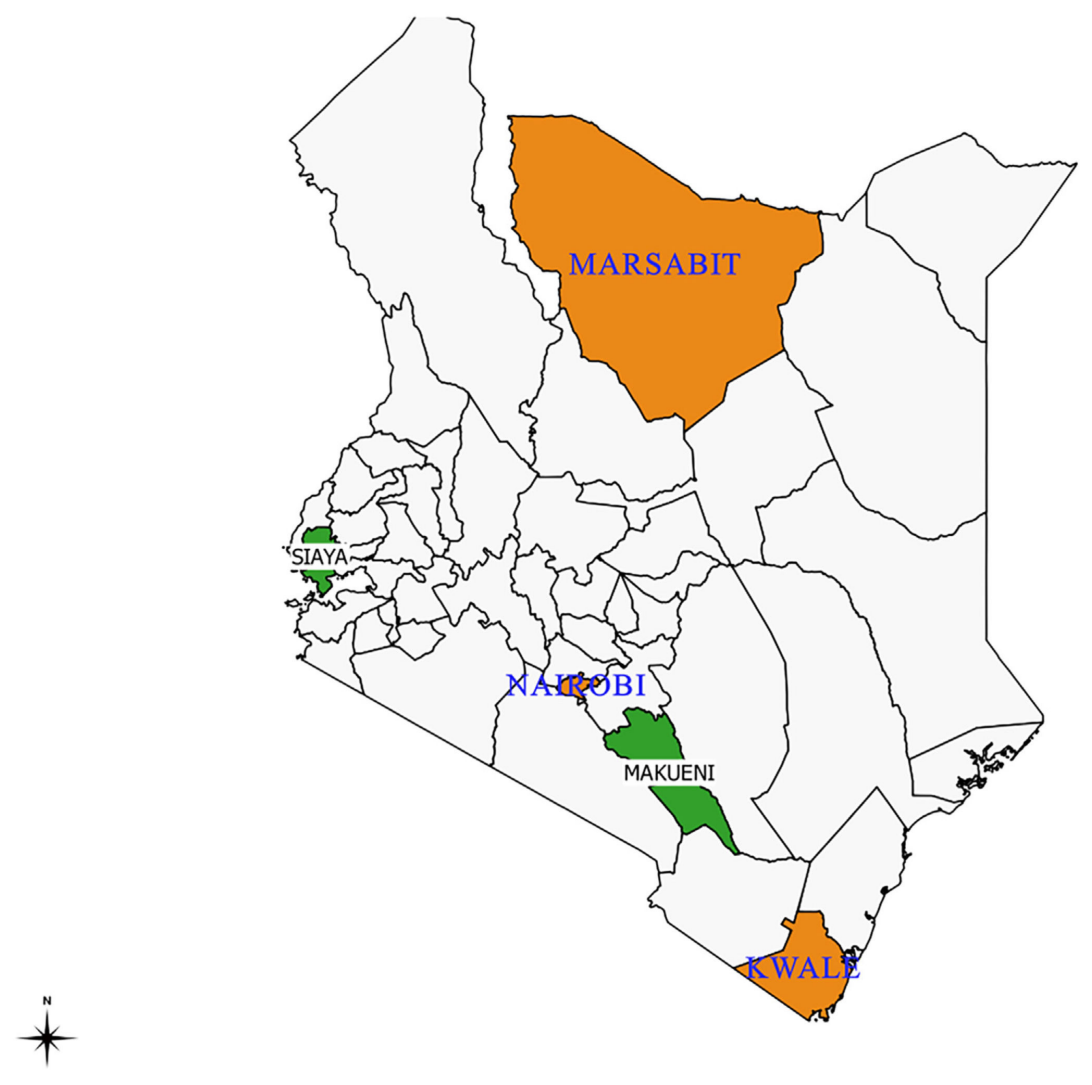
Map of Kenya showing the study counties comprising pilot counties where rabies elimination activities were ongoing (Makueni and Siaya Counties in green) and nonpilot counties (Nairobi, Marsabit and Kwale Counties in orange). (For interpretation of the references to colour in this figure legend, the reader is referred to the web version of this article.)

This survey was conducted in five of 47 counties in Kenya between May and June 2017. To establish the country’s situation in regards to PEP, data over 5 years prior to the interview date were collected. The selection of the study counties was purposive to allow for representation of regions that had started systematic rabies elimination activities (Makueni and Siaya Counties) such as mass dog vaccinations and public health campaigns and for those yet to commence activities (Nairobi, Kwale and Marsabit Counties) (Fig. 1). Nairobi County was included in the study to represent an urban population and to allow for interviews with the main bodies procuring vaccines in the country: the National Vaccine and Immunization program (NVIP), the Kenya Medical Supplies Agency (KEMSA), and the Mission for Essential Drugs and Supplies (MEDS). NVIP is responsible for all the routine vaccines used in the Expanded Program for Immunization (EPI), whereas KEMSA and MEDs mainly support medical supplies including non-routine vaccines for public and private health facilities respectively.

The survey focused on eight thematic areas related to rabies PEP and RIG at the national, county and sub-county levels: program delivery, procurement, requesting, distribution, cold chain and storage, forecasting, monitoring and utilization, and reporting. The details of the information collected under each of the thematic areas are provided in Table 1. The survey questions used were adopted from the PEP logistics and flow questionnaire developed by the World Health Organization and US Centers for Disease Control and Prevention, and implemented across several countries (Appendix Supplementary Table 1).

Separate questionnaires were developed to gather information from relevant persons at NVIP, KEMSA and MEDS at the national level, and directors of health, routine vaccines logisticians, surveillance officers, and pharmacists at the county and sub-county levels. Table 2 provides details on the designation of the 35 persons interviewed for this study, and the specific organizations and health facilities that participated in the survey.

The questionnaires were programmed on the Commcare^®^ phone-based application to allow both electronic capture of quantitative data, and voice recording of the interviews to capture qualitative data. The questionnaires were pre-tested and consent for voice recording and participation in the study obtained from each respondent. The voice records were transcribed by a pair of transcribers allowing for crosschecking of the transcripts.

Ethical clearance for the study was obtained from the Kenya Medical Research Institution Scientific and Ethics Review Unit (KEMRI-SERU protocol No. 3268).

## 3 Results

A total of 35 interview surveys were completed with four respondents drawn from KEMSA, NVIP and MEDS at the national level, 23 respondents at the county and sub-county levels and 8 health care workers at the health facilities.

### 3.1 Overview of the vaccine supply chain structure in Kenya

The supply of routine and non-routine vaccines in Kenya is by three main institutions: NVIP, KEMSA and MEDS. NVIP is the primary government institution responsible for sourcing and supply of routine (EPI) vaccines used in both the public and private health sector. In addition, NVIP supplies non-routine vaccines including typhoid, cholera, and human papilloma virus vaccines. Human rabies vaccines are treated as non-routine vaccines and are not anchored on a specific national program.

KEMSA (a government organization) is the main supplier of rabies vaccines for public health facilities and MEDS (a for-profit private organization) supplies the private health sectors facilities with the vaccines. KEMSA and MEDS receive orders for these vaccines from individual counties, and sometimes directly from health facilities.

NVIP orders for the routine vaccines go through the United Nations Children’s Fund (UNICEF) with facilitated cost estimation, scheduling, procurement and delivery of quality vaccines. Purchase of routine vaccines is financed through the Kenya Government and Gavi, the Vaccine Alliance. NVIP reported procuring small quantities of rabies vaccines but this was rare and dependent on requests received from counties and the availability of funds.

NVIP, KEMSA and MEDS have national depots located centrally for storage of vaccines received before distribution to the counties. At the lower level, NVIP maintains depots at regional and sub-county levels while KEMSA maintains depots at regional level. There are vaccine logisticians placed at health facilities, sub-county and county levels specifically to support the supply and distribution of routine vaccines. At these lower levels, rabies PEP and RIG are handled together with other medical supplies.

### 3.2 PEP and RIG delivery

Before the change from a centralized to a devolved government system, NVIP was the main supplier of rabies vaccines and RIG. This role changed after decentralization with NVIP remaining as the main supplier of routine vaccines, and having a lesser role for rabies biologicals. Our study showed NVIP last procured rabies vaccines in 2015 as an emergency request from counties. As a result, the demand for rabies vaccines from KEMSA and MEDS and their supply of PEP increased (MEDS reported an increase of over 250% in the last five years), making the two bodies the primary suppliers of rabies biologicals. At the County level, 3 of the 5 study counties reported an increase in PEP availability related to increased control of health budgets following devolution, but quantitative information on these changes were not available.

For the 12 months preceding the PEP surveys, all counties reported experiencing stockouts for the rabies vaccines. Availability of rabies biologicals varied by counties. Counties implementing elimination activities (Makueni and Siaya Counties) had shorter stockouts (3.5 weeks and 5 weeks respectively), while Kwale, Nairobi and Marsabit Counties reported stockouts of 36, 32 and 8 weeks respectively. The main reason for stockouts reported was the high cost of PEP, delayed procurement, coupled with a high incidence of dog bites.

RIG was rarely provided, with NVIP and MEDS reporting to have been out of stock for the preceding five years. KEMSA stocked RIG previously but the counties did not order for it, leading to expiry of available stock and losses for KEMSA. Information on the number of vials, type and utilization of RIG was not available. It can however be assumed that use of RIG was negligible, as all respondents at the sub-national level did not know about RIG. Information on the category of bite, that would be important for RIG prioritization, was not recorded.

Rabies PEP vaccine was administered through the intramuscular route in 5 doses (the ‘Essen” regimen - day 0, 3, 7, 14 and 28). None of the counties reported using the intradermal vaccination regimens recommended by WHO.

### 3.3 Rabies PEP forecasting, request and procurement processes

Rabies PEP forecasting is required at three main levels: health facility where PEP is administered to bite patients, sub-county/county level that funds procurement of vaccines and distributes PEP to the health facilities, and at national level to guide quantities ordered and imported into the country. The methods of forecasting at the three levels are different, and there are further differences in the forecasting for routine versus non-routine vaccines.

At the national level, forecasting for routine vaccines uses a combination of consumption data, estimated size of the target population, projected population growth and expected level of vaccine wastage. Whereas this holds true for routine vaccines that do not require counties to pay for the vaccines, the volumes of rabies vaccines ordered are dependent on estimation of the numbers that counties will likely order and pay for.

To determine vendors that will supply the vaccines at the national level, KEMSA uses an open tender system specifying quantities, desired quality and delivery timelines, and inviting local and international vendors to place their bids. Submitted bids are evaluated based on both documentation and quality of pre-delivery samples. MEDS uses a closed system consisting of annual evaluation of existing and prospective suppliers by a technical committee, that may include visiting manufacturing sites.

The forecasting system for non-routine vaccines at county level is less developed as compared to routine vaccines. Rabies procurement at county level is based on consumption data and does not take into account number of bites from suspect rabid dogs – data routinely collected at health facilities through the Ministry of Health District Health Information Systems (DHIS2), or data on patients that did not access PEP at the time of seeking care.

Requests for routine vaccines are done using a standardized vaccine request/order form filled in by sub-county logisticians, aggregated at the county level and submitted to NVIP. Similar online forms are filled at the county level to request for rabies vaccines from MEDS and KEMSA. However, the procurement of rabies vaccines at the county level is irregular and dependent on the county’s allocation of funds towards rabies. Three of the 5 study counties cited financial constraints as the main reason for delays in procuring PEP.

On average, PEP costed counties approximately 8USD per dose to procure from KEMSA and MEDS. In the absence of government sub-sidies for PEP, bite patients paid 10-15 USD per dose at the county level. Costs of PEP to the patient were not standardized and differed by county. In the 4 months preceding the survey, Siaya county reported subsidizing PEP costs by 50% enabling bite patients to pay 5USD per dose. Makueni County provided PEP for free for any resident enrolled in the Universal Health Coverage (UHC) plan. RIG procurement at the national level was erratic and KEMSA reported procuring equine RIG at approximately 8USD per vial between 2014 and 2016. KEMSA had not procured any RIG in the 12 months preceding the survey interview, which was attributed to a lack of demand for the product at the counties. The cost of RIG to patients from the private sector was approximately USD70 per vial.

### 3.4 Vaccine distribution and supply duration

The distribution of routine vaccines from the national level to the counties uses a push mechanism. Vaccines are shipped to the locality of request. Whereas routine vaccines have storage depots located at the county/sub-county headquarters, rabies vaccines do not use these facilities resulting in logistical difficulties of getting them to the health facilities.

Rabies vaccines are instead delivered to the county headquarters, and supplied to the health facilities through a pull distribution system organized around the monthly County Health Management Team (CHMT) meetings. Sub-County health officials attending the meetings collect the vaccines and deliver to the health facilities. Four of five counties reported stocking rabies vaccines only up to the sub-county level. Only Marsabit county reported stocking PEP in some health facilities with reported high number of bite cases.

**Fig. 2:**
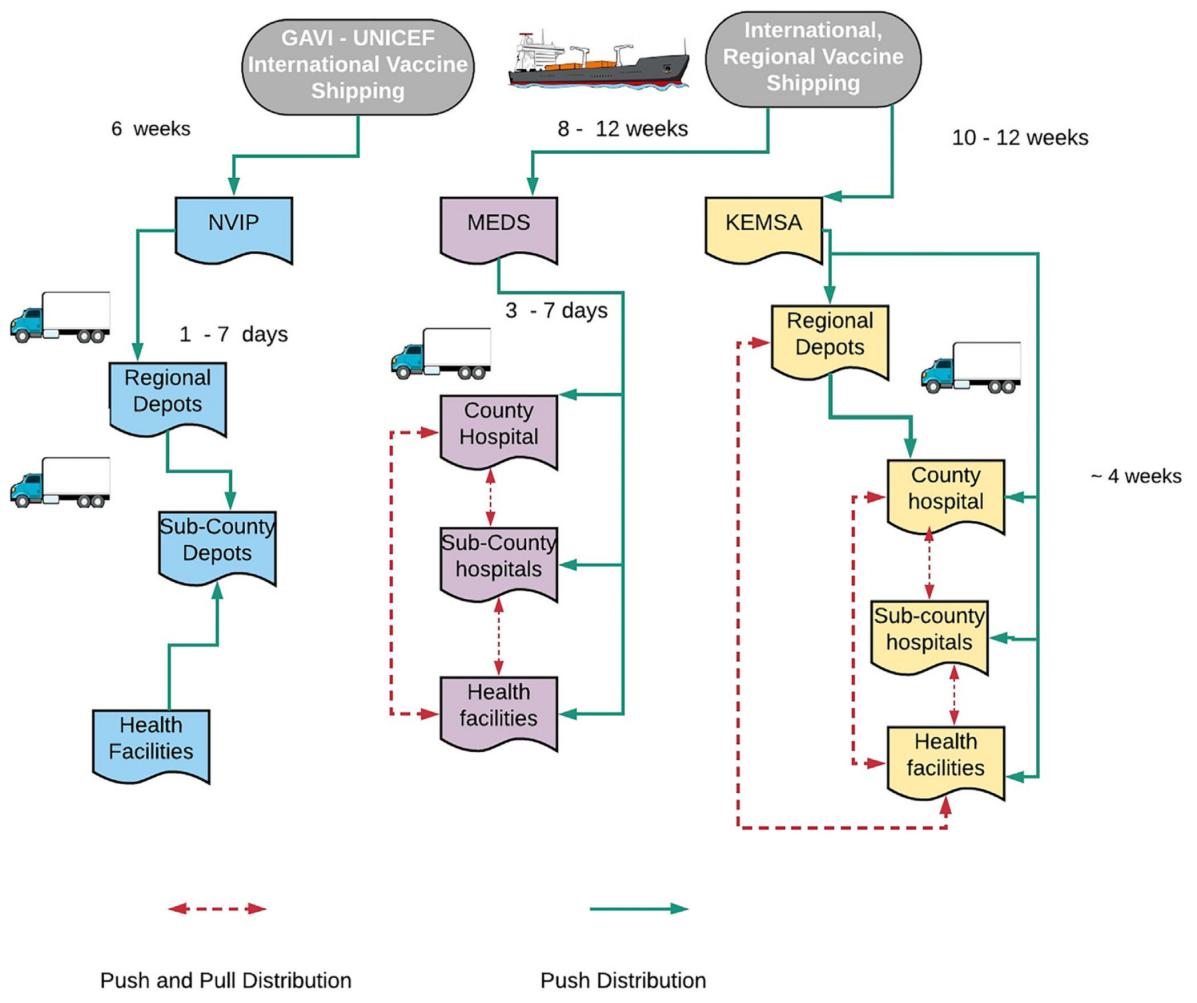
Schematic diagram showing the vaccine distribution system in Kenya.

On average, once ordered, delivery of routine vaccines from international distributors took 6 weeks to get to NVIP, and 8 and 11 weeks to get to MEDS and KEMSA respectively. Supply from the national to the subnational levels took 1 week for routine vaccines and rabies vaccines supplied to private facilities via MEDS versus 4 weeks for supply of public health facilities via KEMSA (Fig. 2).

### 3.5 Availability of cold chain equipment

The non-routine vaccines and routine vaccines do not share the cold chain facilities. Routine vaccines have specific fridges at the sub-county level in more than 50% of the sub-counties in the study counties. Counties reported not having specific fridges for non-routine vaccines at the sub-county level. Only Makueni reported having fridges for non-routine vaccines in six out of 40 health facilities.

### 3.6 Monitoring and evaluation

Monitoring of routine vaccines by NVIP is elaborate and robust, consisting of an electronic system capturing details of stock levels up to the sub-county level, supported by regular field visits and supervision on routine vaccines management at the county and sub-county levels. KEMSA and MEDS have monthly and semiannual stock takes to monitor product movement. In contrast, monitoring of rabies vaccines at the county level is manual and the tools used are not standardized across countries (for routine vaccines, respondents identified the same tool to monitor vaccine usage within counties).

Although rabies PEP requires multiple doses, there was no system in place to ensure dose completion compliance. Patients are advised to complete all five doses and dates are detailed in the patient’s card, however anti-rabies registers were not in use in counties and no follow up is made to patients who do not complete their doses. Data on anti-rabies vaccine wastage is not regularly collected or reported. Adverse events from medicines and vaccines are reported to the Pharmacy and Poisons Board through standardized forms that include information on symptoms experienced, date of symptom onset, and product batch numbers. Table 3 summarizes the similarities and differences between routine and non-routine vaccines by the three main vaccine suppliers and the counties.

## 4 Discussion

This study has shown considerable in-country variability in the availability of rabies vaccines and immunoglobulin, a less robust and inadequate supply system for rabies biologicals operated separately to that used by routine vaccines, use of the intramuscular route for PEP administration as opposed to the dose-saving intradermal route, and a high cost of rabies PEP and RIG to bite patients. Taken together, our results point to a sub-optimal system requiring specific improvements to achieve prompt provision of rabies PEP for persons exposed to rabies.

The large differences in the stockout periods between counties indicate differential prioritization of rabies at the local level. Awareness created in counties implementing rabies elimination activities likely resulted in the increased availability of the vaccines and relatively shorter stockout periods. For the country to achieve the 2030 goal of elimination of human rabies, provision of readily available and affordable PEP across all counties where rabies is endemic, is important. A good example is Thailand which has dramatically reduced human deaths from rabies to below 10 cases per year by educating the public and health care workers, and providing PEP free of charge across the country, before mass dog vaccinations reached the recommended 70% coverage [18]. Ultimately, elimination of human rabies is dependent on eliminating the disease in dogs. Combining the use of dog and human vaccinations during the elimination phase is an effective and cost-effective approach to progressively reducing human deaths from rabies [19].

To increase rabies vaccine availability at health facilities, the current forecasting and stock monitoring requires improvements. Several opportunities to improve the system exist including program changes that would allow rabies vaccines to use the existing more developed supply system used for routine vaccines. Rabies vaccines are demand-driven and mainly required for a targeted and relatively small group, and are therefore unlikely to overload an existing system. Re-introduction of rabies vaccine into the routine vaccine infrastructure as was the case before devolution is therefore not expected to affect the provision of existing vaccines. However, the impact of such a policy on how the system should be monitored [20,21].

A limitation of our study was that we were unable to obtain information from respondents on the number of bite victims receiving vaccine or the number of vials purchased and used. This was because there are no systems for monitoring these numbers and highlights the need for improvement. The dog-bite data routinely collected at health facility level and transmitted through the District Health Information System 2 (DHIS2) health surveillance open resource tool was not utilized in forecasting for rabies vaccines, although it has potential to be used in this way. Additionally, the lack of a monitoring system for dose-completion at health facilities can be improved through the DHIS2 system by including individual-level data as opposed to the current aggregate data collection. Such an improvement in DHIS2 reporting can improve surveillance data for rabies and provide a basis for advocacy to raise the priority of and funding for rabies vaccines [22].

The cost of PEP remains a major barrier to PEP accessibility. Several opportunities to reduce the cost and increase accessibility exist. The first is the adoption of the WHO recommended dosesaving intradermal route for administration of PEP versus the intramuscular route currently in use [11]. Intradermal injections are not new and are routinely used for vaccination against tuberculosis, including in rural areas. Adoption of the ID route requires advocacy to overcome challenges of inertia to use of new methods and off-label use of ID administration before the vaccine manufacturers have updated vial labels. Introduction of animal bite centers could pool bite patients in a region, and integrated bite case management could aid in judicious and appropriate administration of PEP, by confirming rabid dogs and identifying healthy dogs where PEP is not required [23–25]. The second opportunity comes with introduction of universal health coverage (UHC). In Makueni County where UHC is being implemented, patients enrolled in the program do not pay to receive PEP injections. To ensure availability of the vaccines in the context of UHC, proper forecasting and supply systems coupled with adequate financing for the UHC programs are required. Such systems for rabies could benefit from systems developed for routine vaccines supported by Gavi, the Vaccine Alliance. Gavi recently updated their vaccine investment strategy and included rabies vaccines in their list of vaccines prioritized for future support. Gavi support could facilitate the shared use of cold chain and vaccine supply infrastructure and critically would make rabies vaccines available to bite patients at the point of care at no cost.

## 5 Conclusion

Prompt provision of rabies PEP remains a critical strategy to Kenya achieving zero human deaths from rabies by 2030. Integrating the provision of rabies vaccines and immunoglobulin within the health system including using existing infrastructure for routine vaccines, extending the DHIS2 reporting system to cover PEP demand, use and compliance, supporting universal health coverage, better health financing and free provision of PEP, as well as adoption of ID vaccination regimens, and creation of integrated bite management programs can all increase progress towards eliminating human rabies by 2030.

## Supplementary Material

Table S1

## Figures and Tables

**Table 1:** Details of the eight thematic areas covered in the rabies biologicals survey and the data collected.

Thematic areas	Variables
Program delivery	- Availability and changes in PEP demand, supply and logistics of delivery - Cost of PEP to health facilities and bite patients - Information sharing to bite patients on PEP availability, and facilities
Procurement	- Differences between procurement for routine and non-routine vaccines - Organization of the procurement system and PEP types procured - Costs and time taken from procurement to delivery - Frequency of procurement and individuals responsible
Request	- Requesting procedure for PEP at sub-county, county and national levels compared to routine vaccines - Frequency of requests, individuals responsible
Distribution	- Distribution procedures for PEP from national to county to sub-county levels comparison with routine vaccines - Frequency and individuals responsible
Cold chain and storage	- Cold chain and storage facilities for PEP, compared to routine vaccines national to county to sub-county levels
Forecasting	- Methods of quantification and forecasting for PEP - Comparison with routine vaccines - Individuals responsible
Monitoring and utilization	- System for monitoring usage and stocks of PEP - Comparison with routine vaccines - Individuals responsible - PEP stock out periods and wastages - Monitoring of patients to ensure compliance with completion of PEP
Reporting	- Data collection and reporting on PEP use - Reporting of adverse effects and PEP wastage - Individuals responsible and frequency of reporting

**Table 2:** Details of the persons, their designation, institutions, and health facilities that participated in the survey at National, County and sub-County level.

Level	Institution	Designation
National level	National Vaccine Immunization Program (NVIP) Mission for Essential Drugs and Supplies (MEDS) Kenya Medical Supplies Agency (KEMSA)	Manager supply chain, Quality Assurance Manager, National depot manager, Regional depot manager, Program officer
County level	Marsabit, Nairobi, Makueni, Siaya, Kwale	County EPI Logistician, Pharmacist, Surveillance officer, Public Health officer
Sub-county level	Marsabit (Saku sub-county) Nairobi (Kasarani sub-county) Makueni (Kibwezi West and Makueni sub-counties) Siaya (Alego Usonga sub-county) Kwale (Lunga Lunga sub-county)	Sub-county Public Health Officer, sub-county Disease Surveillance officer, sub-county EPI Logistician, sub-county pharmacist
Health facility level	Marsabit and Laisamis sub-county hospitals Makindu and Makueni County Referral Hospital Siaya County Referral Hospital, Lwak Mission Hospital), Kwale (Msambweni County Referral Hospital)	Pharmaceutical technologist Medical officer in-charge Facility clinical officer

**Table 3:** Table detailing similarities and differences between routine and non-routine vaccines by each of the three main suppliers and at the counties.

MEDS		KEMSA	NVIP	Counties
Procurement system	Closed tender	Open tender	Closed tender	Direct order
Main suppliers of vaccines	Sanofi Pasteur	Medisel Kenya Limited, Sai pharmaceuticals Limited, Surgipharm Limited	GAVI	KEMSA, MEDS, NVIP
Supply duration	8 – 12 weeks	10 – 12 weeks	6 weeks	1 – 4 weeks
Frequency of supply	Quarterly/on demand	Quarterly/on demand	Quarterly/on demand	Quarterly/on demand
Institutions distributed to	Counties, non-governmental, community-based and faith-based institutions	Counties, non-governmental organizations, Faith-based institutions, Public institutions	Counties	Sub-counties and health facilities
Distribution time	3 – 7 days	Up to 1 month	3 – 7 days	1 day
Vaccines available	Yellow Fever, Typhoid and Hepatitis B, Rabies	Yellow Fever, Typhoid and Hepatitis B, Rabies	Routine vaccines, Hepatitis B, Yellow Fever, Meningitis, Tetanus etc.	Routine and nonroutine vaccines
Brand of PEP	Verorab	Indirab	Does not procure PEP	Dependent on supplier
Cost per dose to the organization (rabies vaccine)	Not revealed	USD 3 – 12	Not applicable for rabies. Cost varies for other types of vaccine	USD 8 – 12
Cost per dose to the organization (RIG)	Does not supply RIG	USD 7 – 9	Does not supply RIG	Does not procure RIG
Department/person responsible	Procurement	Procurement	Procurement	Pharmacist – PEP Routine vaccine logistician
Monitoring and evaluation tools used	Electronic (enterprise resource) system	Ledger books and stock management tools	Ledger books and stock management tools	Ledger books and improvised registers

## References

[1] Abela-Ridder B, Knopf L, Martin S, Taylor L, Torres G, De Balogh K (2016). 2016: the beginning of the end of rabies?. Lancet Glob Heal.

[2] Lembo T, Hampson K, Kaare MT, Ernest E, Knobel D, Kazwala RR (2010). The feasibility of canine rabies elimination in Africa: Dispelling doubts with data. PLoS Negl Trop Dis.

[3] Knobel DL, Cleaveland S, Coleman PG, Fevre EM, Meltzer Ml, Miranda MEG (2005). Re-evaluating the burden of rabies in Africa and Asia. Bull World Health Organ.

[4] Lankester F, Hampson K, Lembo T, Palmer G, Taylor L, Cleaveland S (2014). Infectious disease. Implementing Pasteur’s vision for rabies elimination. Science.

[5] Hampson K, Coudeville L, Lembo T, Sambo M, Kieffer A, Attlan M (2015). Estimating the global burden of endemic canine rabies e0003709. PLoS Negl Trop Dis.

[6] Scott TP, Coetzer A, De Balogh K, Wright N, Nel LH (2015). The Pan-African Rabies Control Network (PARACON): A unified approach to eliminating canine rabies in Africa. Antiviral Res.

[7] Davlin SL, VonVille HM (2012). Canine rabies vaccination and domestic dog population characteristics in the developing world: A systematic review. Vaccine.

[8] Jibat T, Hogeveen H, Mourits MCM (2015). Review on dog rabies vaccination coverage in Africa: A question of dog accessibility or cost recovery?. PLoS Negl Trop Dis.

[9] Cleaveland S, Thumbi SM (2018). Proof of concept of mass dog vaccination for the control and elimination of canine rabies.

[10] Dodet B, Durrheim DN, Rees H (2014). Rabies: underused vaccines, unnecessary deaths. Vaccine.

[11] World Health Organization (2018). Rabies vaccines: WHO position paper, April 2018 - Recommendations. Vaccine.

[12] Briggs DJ (2012). The role of vaccination in rabies prevention. Curr Opin Virol.

[13] Hampson K, Dobson A, Kaare M, Dushoff J, Magoto M, Sindoya E (2008). Rabies exposures, post-exposure prophylaxis and deaths in a region of endemic canine rabies. PLoS Negl Trop Dis.

[14] Dimaano EM, Scholand SJ, Alera MTP, Belandres DB (2011). Clinical and epidemiological features of human rabies cases in the Philippines: A review from 1987 to 2006. Int J Infect Dis.

[15] Permpalung N, Wongrakpanich S, Korpaisarn S (2013). Trend of human rabies prophylaxis in developing countries: Toward optimal rabies immunization. Vaccine.

[16] Bitek A, Eric O, Nanyingi M, Muthiani Y, Mathew M, Muriithi R A hundred years of rabies in Kenya and the strategy for its elimination by 2030.

[17] ZDU (2014). Strategic plan for the elimination of human rabies in Kenya.

[18] Wilde H, Ghai S, Hemachudha T (2017). Rabies: Still a silent killer targeting the poor. Vaccine.

[19] WHO Rabies Modelling Consortium (2019). The potential effect of improved provision of rabies post-exposure prophylaxis in Gavi-eligible countries: a modelling study.

[20] Assi TM, Rookkapan K, Rajgopal J, Sornsrivichai V, Brown ST, Welling JS (2012). How influenza vaccination policy may affect vaccine logistics. Vaccine.

[21] Levin A, Wang SA, Levin C, Tsu V, Hutubessy R (2014). Costs of introducing and delivering HPV vaccines in low and lower middle income countries: inputs for GAVI policy on introduction grant support to countries. PLoS ONE.

[22] Scott TP, Coetzer A, Fahrion AS, Nel LH (2017). Addressing the disconnect between the estimated, reported, and true rabies data: the development of a regional African rabies bulletin. Front Vet Sci.

[23] Bharti OK, Madhusudana SN, Kale A, Gaunta PL, Chaudhry LS, Kumar J (2015). Success story of a low cost intra-dermal rabies vaccination (IDRV) cliniclessons learnt over five years of 12,000 patient vaccinations “without failure” at DDU Hospital Shimla, Himachal Pradesh, India.

[24] van de Burgwal LHM, Neevel AMG, Pittens CACM, Osterhaus ADME, Rupprecht CE, Claassen E (2017). Barriers to innovation in human rabies prophylaxis and treatment: A causal analysis of insights from key opinion leaders and literature. Zoonoses Public Health.

[25] Wallace RM, Reses H, Franka R, Dilius P, Fenelon N, Orciari L (2015). Establishment of a canine rabies burden in haiti through the implementation of a novel surveillance program. PLoS Negl Trop Dis.

